# Planar Position Sensor Based on Mono Sensing Electrode and Hybrid-Frequency Excitation

**DOI:** 10.3390/s16050691

**Published:** 2016-05-13

**Authors:** Hongxiang Yu, Yu Zhang, Mengfeng Shen, Hongli Zhang, Zhao Gao, Dongyun Wang

**Affiliations:** College of Engineering, ZheJiang Normal University, Jinhua 321004, China; zjnuzy@zjnu.cn (Y.Z.); shenmengfeng@163.com (M.S.); tz_zhl@163.com (H.Z.); gz@zjnu.cn (Z.G.); zsdwdy@zjnu.cn (D.W.)

**Keywords:** planar position, capacitive sensor, mono-electrode, hybrid-frequency

## Abstract

A new way of measuring planar position for micrometric and sub-micrometric applications is presented with a mono sensing electrode and hybrid-frequency excitation. The sensing theory and operation principle are described and summarized, and a printed circuit board (PCB) sensor prototype is built and tested. It is shown by the experimental results that a very simple structure and geometric relationship are achieved. Meanwhile, displacement sensitivity on an order of 1.50 mV per micron and measurement repeatability better than 0.002 mm are easily fulfilled for a square zone of 256 mm^2^, making it a valuable alternative measurement device candidate for flexible and low-cost planar position detection.

## 1. Introduction

Micrometric and sub-micrometric positioning measurement plays an important role in precision metrology and manufacturing applications [1,2]. Their uses include linear positioning measurement, planar positioning measurement, and space positioning measurement, *etc.* [3,4]. Hence many types of sensor devices have emerged, such as laser interferometers, grating scales, and capacitive transducers [5,6,7]. However, most of them were originally designed for single dimension measurement, for instance, in a linear positioning mechanism or in a rotary servo motor. To perform an inspection in the 2D plane, the accumulation of single dimension sensors is inevitable in most cases, which leads to complicated structures and cumbersome size. Particularly, due to the orthogonal deviation of assembly, such combined apparatus can easily introduce Abbe and cumulative errors into the measurement results [8], so alternative methods of measuring planar position continue to be an open field of research of high interest [9]. Among these methods, planar gratings, optical image processing, and capacitive coupling strategies can serve as a relevant options, both for theoretical research and for technological applications. For example, in [10], two planar gratings were diagonally mounted on a planar moving stage for detecting planar position and deflection on the nanoscale. In [11,12], laser speckle patterns and diffuse-reflectivity images are analyzed for measuring 2D displacement with high dynamics, and in [13], a capacitive transducer utilizing eight sensing electrodes was developed for the detection of planar displacements. However, most planar grating devices involve high costs, despite their high resolution, and the existing capacitive coupling methods generally need to solve complex capacitance signals generated by multiple sensing electrodes. For the growing demand of absolute position measurement in the 2D plane, sensor devices of micrometric and sub-micrometric resolution with simple structures and low-cost have not been experimentally demonstrated.

This paper introduces a new approach to absolute planar position detection by employing a mono sensing electrode and a hybrid-frequency excitation scheme. The physical theory and operation principle are described and summarized. In addition, experiments are carried out with a PCB sensor prototype, to verify the feasibility and to demonstrate the advantages.

## 2. Theory and Principle

**Figure 1:**
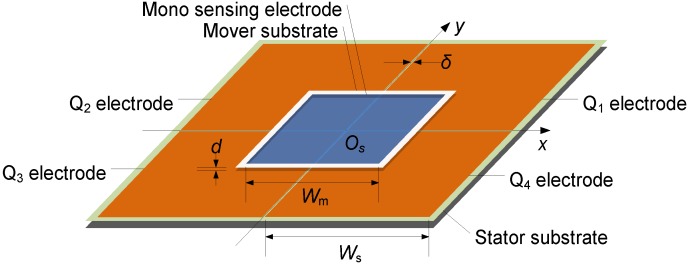
Schematic of the proposed planar position measurement method.

The proposed sensor design measures planar position by reading the amplitudes and phasors of dual AC voltage signals with different frequency. Figure 1 shows the basic structure of the new design. In order to establish an absolute measurement reference, a stator substrate is symmetrically divided into four quadrants designated as *Q*_1–4_ by the coordinate system *O_s_xy*. In each quadrant, a square emission electrode is assigned with sides *W_s_*, and all four of the quadrant electrodes are isolated from each other by a clearance of δ to ensure electric isolation. On the mover substrate, a mono sensing electrode with sides *W_m_* is arranged, meeting the condition that *W_m_* is less than *W_s_*. During position inspection, the mover is maintained to be parallel and aligned with the stator by a steady air gap *d*, while the mono sensing electrode concurrently covers the four emission electrodes.

**Figure 2:**
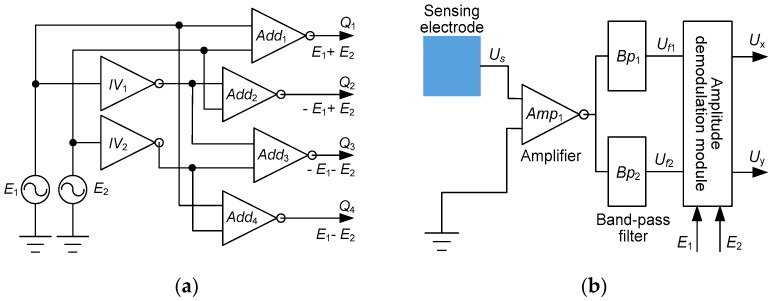
Electronics circuit scheme. (**a**) Driving circuit for the four quadrant electrodes; (**b**) Signal processing circuit for the mono sensing electrode.

For the synchronous perception of *x*-axis position and *y*-axis position, Figure 2a demonstrates a hybrid-frequency excitation circuit for the four quadrant electrodes, in which AC voltage sources *E*_1_ and *E*_2_ are introduced with different frequencies *f*_1_ and *f*_2_, and analog inverters *IV*_1_, *IV*_2_ are utilized in conjunction with four analog adders *Add*_1–4_. Figure 2b shows a signal processing circuit for the reading of planar position, where mono-sensing signal *U_s_*, coupled from the *Q*_1–4_ electrodes, is firstly buffered by a high input-impedance amplifier *Amp*_1_. Then the harmonic components of frequency *f*_1_ and frequency *f*_2_, *U_f_*_1_ and *U_f_*_2_, are extracted by band-pass filters *Bp*_1_ and *Bp*_2_, respectively. Hence dual DC outputs *U_x_* and *U_y_*, indicating the mover’s planar coordinates (*x*,*y*) in coordinate system *O_s_xy*, are derived through an amplitude demodulation module.

**Figure 3:**
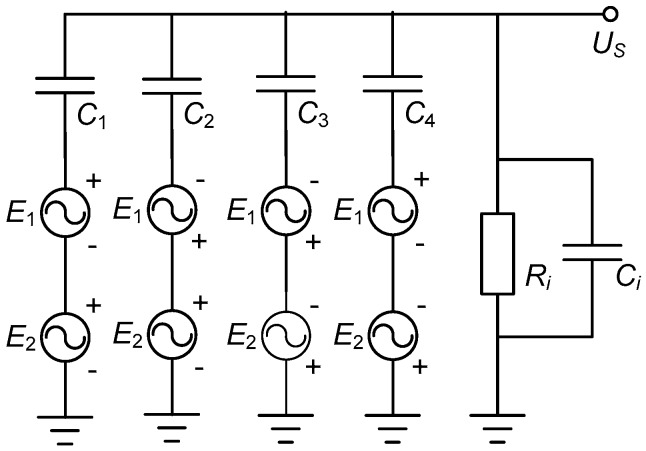
Equivalent circuit of the capacitive coupling process.

To summarize the operation principle, Figure 3 illustrates an equivalent circuit of the capacitive coupling process; in which *C*_1–4_ sequentially indicates the capacitors formed by the mono sensing electrode and the four emission electrodes located in *Q*_1–4_. *R_i_* stands for the input resistance of *Amp*_1_, *C_i_* stands for its input capacitance. Given time functions of *E*_1_ and *E*_2_ as:

(1) $$\left\{ \begin{array}{l} e_{1}(t)=u_{e}\sin(2\pi f_{1}t)\\ e_{2}(t)=u_{e}\sin(2\pi f_{2}t) \end{array}\right.$$

The harmonic component *U_f_*_1_ can be solved in vector form as:

(2) $${\overset{•}{U}}_{f1}=\frac{u_{e}k_{a}k_{f1}(C_{1}+C_{4}-C_{2}-C_{3})}{(1+{\left(2\pi f_{1}R_{i}C_{total}\right)}^{-2})C_{total}}\left(1+j\frac{1}{2\pi f_{1}R_{i}C_{total}}\right)$$

where *C_total_* means the sum of *C*_1–4_ and *C_i_*, *k_a_* indicates the gain of *Amp*_1_, and *k_f_*_1_ stand for the attenuation of *B_p_*_1_, respectively. Similarly, *U_f_*_2_ can be deduced as:

(3) $${\overset{•}{U}}_{f2}=\frac{u_{e}k_{a}k_{f2}(C_{1}+C_{2}-C_{3}-C_{4})}{(1+{\left(2\pi f_{2}R_{i}C_{total}\right)}^{-2})C_{total}}\left(1+j\frac{1}{2\pi f_{2}R_{i}C_{total}}\right)$$

Considering the high input-impedance of *Amp*_1_ and the common excitation frequency for a capacitive transducer, the imaginary parts of Equations (2) and (3) are far less than their real parts. In the case of 10^9^ Ω for *R_i_*, 100 kHz for *f*_1,2_, and 1 pF for *C_total_*, imaginary part will be only 0.16% of the real part for both *U_f_*_1_ and *U_f_*_2_. Consequently, Equations (2) and (3) are approximated as:

(4) $$\left\{ \begin{array}{l} {\overset{•}{U}}_{f1}≅\frac{u_{e}k_{a}k_{f1}\left(C_{1}+C_{4}-C_{2}-C_{3}\right)}{C_{total}}\\ {\overset{•}{U}}_{f2}≅\frac{u_{e}k_{a}k_{f2}\left(C_{1}+C_{2}-C_{3}-C_{4}\right)}{C_{total}} \end{array}\right.$$

It is evident in Equation (4) that *U_f_*_1_ is an amplitude modulated signal of capacitance pairs *C*_1,4_ and *C*_2,3_, while *U_f_*_2_ is the amplitude modulated result of *C*_1,2_ and *C*_3,4_. According to the geometries declared in Figure 1, Equation (4) is furtherly reduced by replacing *C*_1–4_ with the Ideal Plate Capacitance Formula, hence expressed as:

(5) $$\left\{ \begin{array}{l} {\overset{•}{U}}_{f1}=\frac{2u_{e}k_{a}k_{f1}}{\left(w_{m}-\delta \right)+\frac{dC_{i}}{\left(w_{m}-\delta \right)\varepsilon_{0}}}x\\ {\overset{•}{U}}_{f2}=\frac{2u_{e}k_{a}k_{f2}}{\left(w_{m}-\delta \right)+\frac{dC_{i}}{\left(w_{m}-\delta \right)\varepsilon_{0}}}y \end{array}\right.$$

where ε_0_ represents the permittivity of the air gap *d*. Therefore, *U_f_*_1_ and *U_f_*_2_ behave as two linear functions of planar coordinates (*x*, *y*), since the remaining parameters can be treated as constants for a specific sensor device. Accordingly, the planar positon of the mover can be determined by deriving the amplitudes of *U_f_*_1_ and *U_f_*_2_ and by judging their in-phase or reversed-phase status *versus* *E*_1_ and *E*_2_, respectively. Such a function is performed by an amplitude demodulated module, which belongs to the previously mentioned signal processing circuit, as shown in Figure 2b.

To sum up, we can synthesize the expressions of amplitude demodulated outputs *U_f_*_1_ and *U_f_*_2_ as follows:

(6) $$\left\{ \begin{array}{l} U_{x}=\frac{2u_{e}k_{a}k_{f1}k_{d}}{\left(w_{m}-\delta \right)+\frac{dC_{i}}{\left(w_{m}-\delta \right)\varepsilon_{0}}}x\\ U_{y}=\frac{2u_{e}k_{a}k_{f2}k_{d}}{\left(w_{m}-\delta \right)+\frac{dC_{i}}{\left(w_{m}-\delta \right)\varepsilon_{0}}}y \end{array}\right.$$

where *k_d_* indicates the gain of amplitude demodulated module.

As for the effective measurement range, it is determined by the basic theory of capacitive coupling, of which the mono sensing electrode must cover the four quadrants’ electrodes concurrently. It implies that the transition of mono sensing electrode is restricted by:

(7) $$\left\{ \begin{array}{l} \left|x\right|\leq \frac{w_{m}-\delta }{2}\\ \left|y\right|\leq \frac{w_{m}-\delta }{2} \end{array}\right.$$

In fact, to reduce fringing effects, a smaller measurement range is much preferred in practice compared with the maximum margin as defined in Equation (7).

## 3. Prototype and Experimental Setup

**Figure 4:**
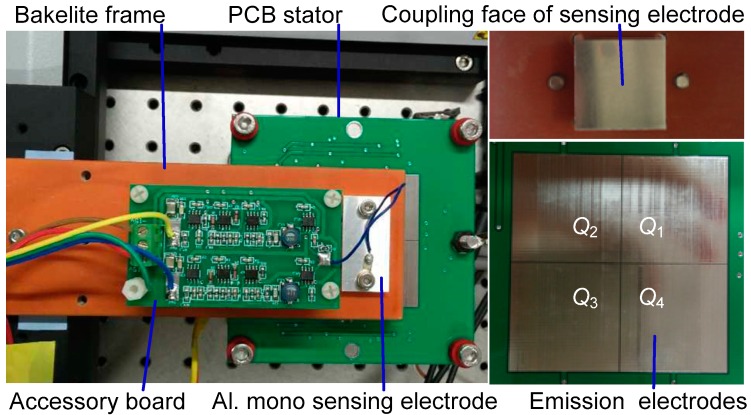
Photograph of PCB sensor prototype.

To verify the feasibility and to demonstrate the advantages of the new sensor design, a laboratory prototype was built with a PCB stator, aluminum mono sensing electrode, and an accessory board for sensing signal processing. On the PCB stator, four emission electrodes are arranged with sides of 30 mm and 0.2 mm clearance to each other. On the mover assembly, a mono sensing electrode with 18 mm side-length is fixed together with the accessory board on a Bakelite frame. Figure 4 shows a photograph of the sensor prototype.

**Figure 5:**
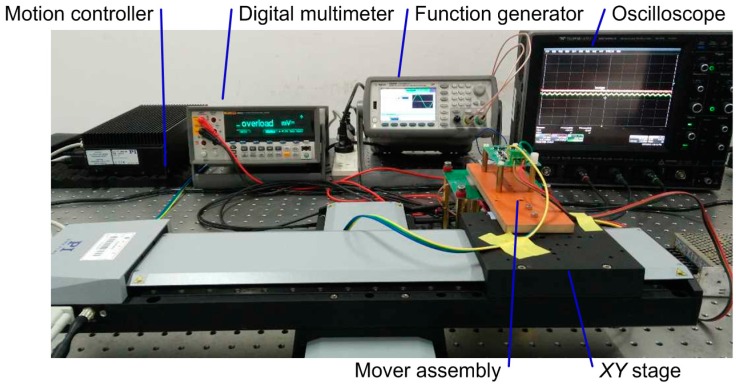
Configuration of the test bench.

The configuration of the test bench for our experimental investigations is shown in Figure 5. It mainly consists of a *XY* stage for operating the mover assembly, a function generator to supply *E*_1_ and *E*_2_, an oscilloscope to probe the signals, a digital multimeter for origin searching, and a motion controller for automatic planar scanning. The *XY* stage (M-531.DD1, Physik Instrumente, Karlsruhe, Germany) and the PCB stator are mounted on a vibration isolator table (T1220Q, Thorlabs, Newton, NJ, USA). The *XY* stage has a motion resolution of 0.0001 mm and a positioning accuracy of 0.00025 mm. Moreover, the motion controller (Physik Instrumente C-884.4D) is capable of synchronous signal acquisition during scanning operation. Before experimental inspection, the planar scanning area is programmed to be a square zone of 256 mm^2^, scanning speed is set to 20 mm/s, sampling interval for *U_x_* and *U_y_* is set as 0.25 mm, and the origin for planar scanning operation is positioned by searching the zero output locations of *U_x_* and *U_y_* in x-axis direction and in y-axis direction. The hybrid-frequency excitation sources of *E*_1_ and *E*_2_ are adjusted to 100 kHz and 950 kHz with the same amplitude of 5 Vpp, and the air gap is adjusted to 1 mm.

## 4. Results and Discussion

**Figure 6:**
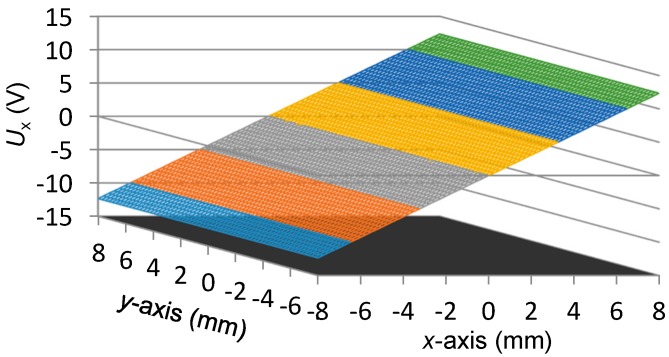
Scanning result of *U_x_*.

**Figure 7:**
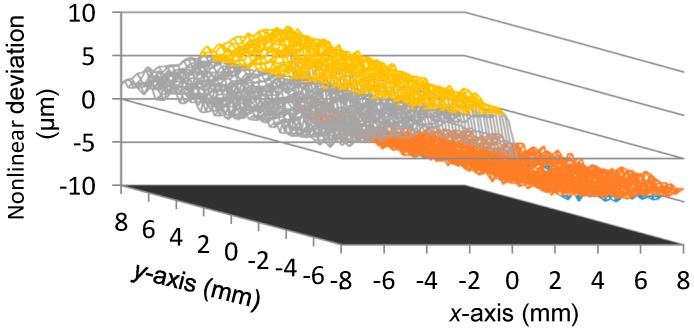
Nonlinear deviations of *U_x_*.

**Figure 8:**
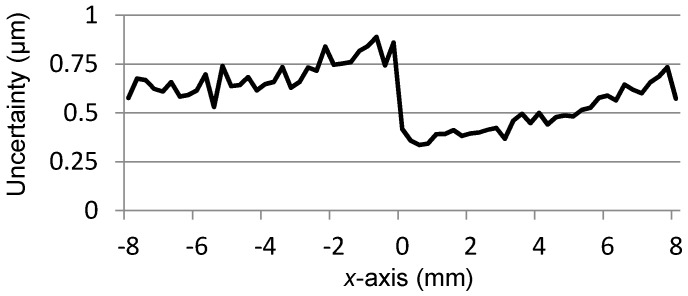
Uncertainty of *x*-coordinate readings.

Figure 6 shows the scanning result of *U_x_*. Figure 7 demonstrates the nonlinear deviation of *U_x_* for the reading of x-coordinate. From Figure 6 and Figure 7, we observe the following:

1. The peak-to-peak value of *U_x_* is about 24.7139 V, thus the displacement sensitivity for the *x*-axis position is about 1.54 mV per micron. The variation of *U_x_* is almost linearly related to the *x*-axis position, however, a nonlinear deviation with a level of 0.0087 mm to −0.0058 mm exists in *U_x_*, which is approximately 0.11% of the full measurement range in the *x*-axis direction.
2. To evaluate the independence of *U_x_* with respect to the transition that occurs in the *y*-axis direction, for each scanning position in the *x*-axis, the outputs of *U_x_* at all the 65 positions of the *y*-axis, as shown in Figure 6, are adopted for the calculation of the uncertainty of *x*-coordinate readings. The utilized method is the standard deviation and the result is presented in Figure 8, where the maximum value is 0.0009 mm. It indicates that the transition of the mover in the *y*-axis direction merely affects the measurement result of the *x*-coordinate.

**Figure 9:**
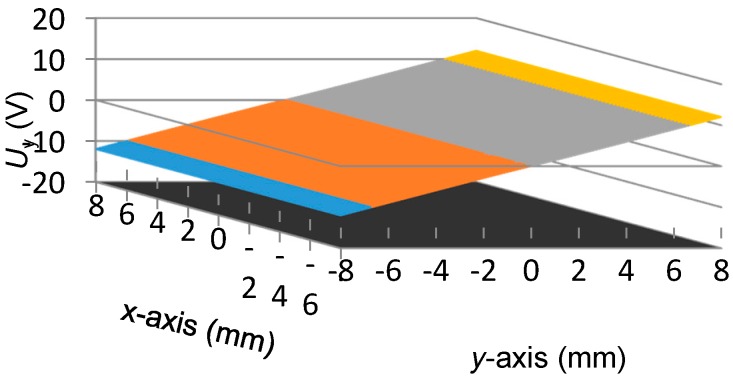
Scanning result of *U_y_*.

**Figure 10:**
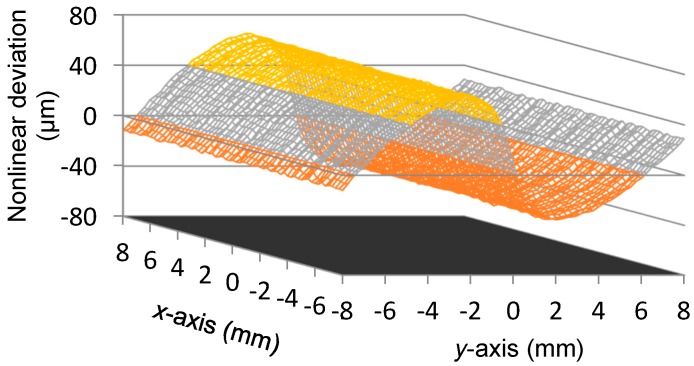
Nonlinear deviations of *U_y_*.

**Figure 11:**
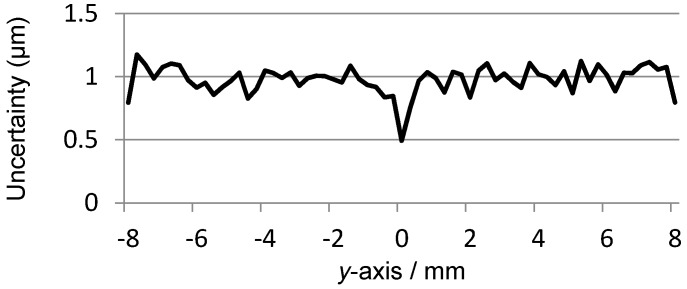
Uncertainty of the *y*-coordinate readings.

Figure 9 gives the scanning result of *U_y_*. Accordingly, Figure 10 illustrates the nonlinear deviation of *U_y_* for the reading of the *y*-coordinate. From Figure 9 and Figure 10, we observe the following:

1. The peak-to-peak value of *U_y_* is 24.0039 V, thus the displacement sensitivity for the *y*-axis position is about 1.50 mV per micron. The variation of *U_y_* is basically linear with respect to the *y*-axis position. A nonlinear deviation with a level of 0.066 mm to −0.037 mm exists in *U_y_*, which is approximately 0.82% of the full measurement range in the *y*-axis direction.
2. Similarly, to evaluate the independence of *U_y_*, for each scanning position in the *y*-axis, the outputs of *U_y_* at all the 65 positions of the *x*-axis, as shown in Figure 9, are adopted for the calculation of the uncertainty of the *y*-coordinate readings. The result is presented in Figure 11, where the maximum value is 0.0012 mm. Thus the transition in the *x*-axis direction displays no evident influence on the measurement results of the *y*-coordinate.
3. Even though the displacement sensitivity and readings uncertainty show no significant differences between the two measurement directions, the nonlinear deviation suffers a sharp rise for the *y*-coordinate readings compared with the response of the *x*-axis. Considering the same signal processing circuit, such a diversity could be caused by the different excitation frequencies assigned for *x*-axis position sensing and for *y*-axis position sensing, namely the 100 kHz and the 950 kHz.

**Figure 12:**
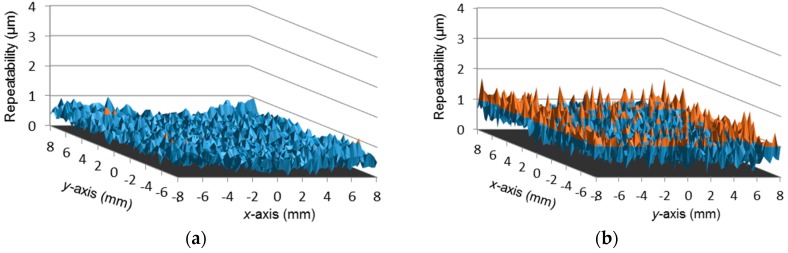
Measurement repeatability. (**a**) Repetitive error of the *x*-coordinate readings; (**b**) Repetitive error of the *y*-coordinate readings.

Finally, the experiment procedure including five repetitive scanning sequences is performed, and the obtained data is adopted to assess the repeatability for planar position measurement by calculating the standard deviation of the *x*-coordinate readings and *y*-coordinate readings at each of the scanning points. Figure 12a shows the repetitive error distribution of the *x*-coordinate readings, where the peak value is 0.0012 mm and the average in the full square zone is 0.0004 mm. Figure 12b presents the repetitive error distribution of the y-coordinate readings, in which the peak is 0.0018 mm and the average is 0.0007 mm.

Therefore, it can be concluded that the newly developed sensor design is capable of satisfying the requirements of micrometric planar position measurement in the millimeter range. On the other hand, reading quality may be further enhanced by reducing the measurement range or by improving the dynamic properties of the interface electronics.

## 5. Conclusions

In conclusion, a new capacitive planar position sensor design is proposed. Its operation principle is summarized, and the general performance is investigated using a laboratory built PCB prototype. Based on the experimental results, the implemented mono sensing electrode and the hybrid-frequency excitation strategy are proved to be an effective method for the simplification of sensor structure and for the suppression of cross interference in 2D position measurement. Within a square zone of 256 mm^2^, the prototype achieves a displacement sensitivity of about 1.50 mV per micron and measurement repeatability better than 0.002 mm. In addition, due to the large dimensions and the strong coupling signal; the mono sensing electrode can be mounted separately from the accessory board. Thus we suggest that this research provides a valuable alternative solution for flexible and low-cost planar position detection applications on the micrometric and sub-micrometric scale.

## Abbreviations

The following abbreviations are used in this manuscript:

PCB	Printed circuit board
2D	Two dimension
AC	Alternative current
DC	Direct current
